# Expanded risk groups help determine which prostate radiotherapy sub-group may benefit from adjuvant androgen deprivation therapy

**DOI:** 10.1186/1748-717X-3-8

**Published:** 2008-04-18

**Authors:** Matthew Beasley, Scott G Williams, Tom Pickles, The BCCA Prostate Outcomes Unit

**Affiliations:** 1 British Columbia Cancer Agency, Vancouver, Canada; 2 Peter MacCallum Cancer Centre, Melbourne, Australia

## Abstract

**Purpose:**

To assess whether an expanded (five level) risk stratification system can be used to identify the sub-group of intermediate risk patients with prostate cancer who benefit from combining androgen deprivation therapy (ADT) with external beam radiotherapy (EBRT).

**Materials and methods:**

Using a previously validated 5-risk group schema, a prospective non-randomized data set of 1423 men treated at the British Columbia Cancer Agency was assessed for the primary end point of biochemical control (bNED) with the RTOG-ASTRO "Phoenix" definition (lowest PSA to date + 2 ng/mL), both with and without adjuvant ADT. The median follow-up was 5 years.

**Results:**

There was no bNED benefit for ADT in the low or low intermediate groups but there was a statistically significant bNED benefit in the high intermediate, high and extreme risk groups. The 5-year bNED rates with and without ADT were 70% and 73% respectively for the low intermediate group (p = non-significant) and 72% and 58% respectively for the high intermediate group (p = 0.002).

**Conclusion:**

There appears to be no advantage to ADT where the Gleason score is 6 or less and PSA is 15 or less. ADT is beneficial in patients treated to standard dose radiation with Gleason 6 disease and a PSA greater than 15 or where the Gleason score is 7 or higher.

## Background

Androgen deprivation therapy (ADT) has a proven role in the treatment of metastatic prostate cancer. Some groups of patients undergoing external beam radiotherapy (EBRT) for localized prostate cancer also benefit from adjuvant ADT. An EORTC trial randomized patients with T1–2 high grade or T3–4 N0–1 prostate cancer to either radiotherapy alone or with 3 years of ADT and showed an improved overall survival at 5 years[1]. The Trans-Tasman Radiation Oncology Group 96.01 trial randomized patients with T2b-T4 N0 disease to radiotherapy alone or with 3 or 6 months ADT. There was an improvement in disease free survival for both ADT arms compared to EBRT alone [2].

Sub-division of prostate cancer patients into risk groups can be used to guide management decisions based on their risk of relapse. The National Comprehensive Cancer Network (NCCN) classifies three risk groups: Low risk (T1-T2a, Gleason score ≤ 6 and PSA ≤ 10 ng/ml), Intermediate risk (T2b-T2c or Gleason score 7 or PSA 10.1–20 ng/ml) and High risk (≥ T3a or Gleason 8–10 or PSA > 20 ng/ml) [3]. When localized prostate cancer is divided into three risk groups, an improvement in the biochemical control rate (bNED) at 5 years has been documented in the intermediate and high risk groups by the addition of neo-adjuvant ADT to EBRT [2,4]. However, a high heterogeneity of outcomes within the intermediate risk patient group has been demonstrated [5]. As ADT causes potentially significant morbidity, it would be desirable to identify within the intermediate risk group a lower risk sub-group who can avoid ADT without compromising cancer control. A five-level risk stratification system with expanded intermediate risk divisions has previously been developed using recursive partitioning analysis and externally validated [6] (see Table 1). The aim of the present study is to determine if these five patient subgroups can better identify those who benefit from the combination of ADT with EBRT.

**Table 1:** Five Level Risk Stratification for prostate cancer. [6]

	Risk Factor		
Risk Group	PSA	Gleason	T-Stage
1. Low	<7.5	≤ 6	any
2. Low-intermediate	7.5–15	≤ 6	any
3. High-intermediate	15–20	≤ 6	any
	≤ 10	≥ 7	any
4. High	20–30	≤ 6	any
	10–20	≥ 7	any
5. Extreme	>20	≥ 7	any
	>30	≤ 6	any

## Methods

A prospective non-randomized patient data set was analyzed, comprising 1583 men treated with EBRT between 1994 and 2001 identified from the Prostate Cohort Outcomes Initiative Database of the British Columbia Cancer Agency (BCCA). After exclusions descried below, 1423 men were available for analysis. All patients received radical EBRT with photon irradiation and CT planning. Those treated with hypofractionated radiation (50–55 Gy in 20 fractions, n = 133) were excluded, as were those who had neoadjuvant ADT of duration <2 months or >12 months, n = 24. Three-dimensional conformal radiotherapy was used from 1998. The median dose administered was 66 Gy (range 66 – 72 Gy) in 2 Gy fractions. Most patients were treated with small volumes to the prostate alone but 177 patients also had a first phase with whole pelvic radiotherapy. Three patients enrolled in a study of ADT versus ADT and EBRT (National Cancer Institute of Canada PR3 study) were excluded.

Patients with higher risk cancers were often selected for combined therapy with neoadjuvant ADT and radiation, but there was no formal policy governing this until 1997. Additionally, because of waiting lists in the early 1990's men with lower risk cancers were also given ADT. Prior to 1997, ADT was delivered by a combination of low-dose stilboestrol (0.1 mg) and cyproterone acetate (50 mg), which has been shown to provide castrate levels of testosterone [7], subsequently LHRH agonist injections (with initial anti-androgen to suppress any androgen flare) were used. Total androgen blockade was not the institutional policy and was used in less than 5% of patients. ADT use was mainly neo-adjuvant until 1997. In 1997, when data was presented showing a benefit from extended adjuvant ADT in high risk patients [8], the BCCA published guidelines [9] and thereafter adjuvant ADT was added to our prior neoadjuvant practice, for an increased overall duration of ADT.

Generally, patients were seen every 6 months for 3 years, then annually for 3 years, then every 2 years. At each visit clinical examination, PSA and testosterone assays, and toxicity were scored. Follow-up is timed from the completion of radiation therapy. Data for all patients was entered prospectively into the database. Additional PSA results from other sources, such as general practice requests, were also incorporated into the database. Institutional ethics review boards approval was obtained for this study.

The standard risk stratification schema was that of the National Comprehensive Cancer Network (NCCN) with three levels: high risk – those with either PSA > 20 ng/mL or T stage T3 or more, or Gleason score 8–10; low risk – those with a PSA <10 ng/mL and T2a or less stage, and GS 6 or less; intermediate risk – all those not high or low risk. Our five level investigational risk schema is shown in Table 1.

The primary endpoint for the study is the absence of biochemical evidence of disease (bNED), which has been shown to be an independent predictor of overall survival when radiotherapy alone has been used to treat prostate cancer [10] and is frequently used as a early end-point in studies of prostate cancer. Patients in this study were assessed for the primary endpoint of biochemical control with the "Phoenix" (lowest PSA to date +2 ng/mL) bNED criterion, as this is robust with or without the use of ADT, unlike the ASTRO definition which is not recommended for studies using ADT[11]. Overall survival (OS) and cause-specific survival (CSS) were obtained from direct data linkage with provincial and national death registries. Assigned deaths due to prostate cancer in the absence of known metastatic relapse, or death from another cause in the presence of known metastatic prostate cancer, were checked manually by chart review.

Comparison of patient characteristics was made using chi-squared tests for categorical data and Kruskal-Wallis tests for non-parametric tests. Time to end-point events was derived using the Kaplan-Meier technique with corresponding log-rank tests of significance. Patients were censored at the time of last clinical follow-up. P values of <0.05 were considered significant.

## Results

The median follow-up was 5 years for biochemical status, (range 1 month – 11 years) and 7.7 years for survival, range 3 months – 12 years). Patient characteristics by expanded and NCCN risk groups are shown in table 2. ADT was used more frequently and for progressively longer durations in higher risk groups.

**Table 2:** Patient characteristics from the Prostate Cohort Outcomes Initiative Database of the British Columbia Cancer Agency (BCCA) sorted by NCCN risk groups [3] and 5-level risk stratification. [6]

	Expanded Risk Groups (Williams, Duchesne,2006)					NCCN Risk Groups		
	Low (n = 317)	Low-intermediate (n = 293)	Intermediate (n = 329)	High (n = 241)	Extreme (n = 230)	Low (n = 229)	Intermediate (n = 497)	High (n = 677)

PSA								
Median	4.9	10.1	7.7	15.8	36	6.1	9.8	16
Range	0.2–7.5	7.6–15	0.3–20	10.1–30	20–250	0.2–10	0.3–20	0.5–250
T stage								
T1	70	77	46	22	23	116	82	40
T2	187	151	150	111	62	113	415	134
T3	56	58	126	97	121	0	0	462
T4	2	3	5	7	15	0	0	32
missing	2	4	2	4	9	0	0	9
Age								
Median	71	72	71	71	69	71	72	70
Age range	46–84	50–82	49–86	47–85	48–82	54–84	50–86	46–84
EBRT dose								
Median (Gy)	66	66	66	66	66	66	66	66
Range (Gy)	66–70	66–70	66–72	66–72	66–70	66–70	66–72	66–72
Gleason score								
6 or less	317	293	74	61	44	229	308	246
7	0	0	175	135	109	0	189	227
8 or more	0	0	80	45	77	0	0	205
ADT rate [Neoadjuvant alone, neoadjuvant-adjuvant]	19.2% [72%, 28%]	23.5% [55%, 45%]	51.7% [39%, 61%]	62.2% [36%, 64%]	79.6% [41%, 59%]	13.5% [87%, 13%]	25.4% [47%, 53%]	69.7% [40%, 60%]
Duration of ADT (neoadjuvant ADT) mean, [total SD] in months)	8.3 (1.1) [6.0]	10.8 (1.5) [7.3]	15.4 (3.2) [13.9]	15.4 (4.0) [13.4]	17.3 (4.7) [16.7]	7.0 (0.8) [3.4]	11.9 (1.6) [12.0]	16.2 (4.3) [14.5]

**Figure 1:**
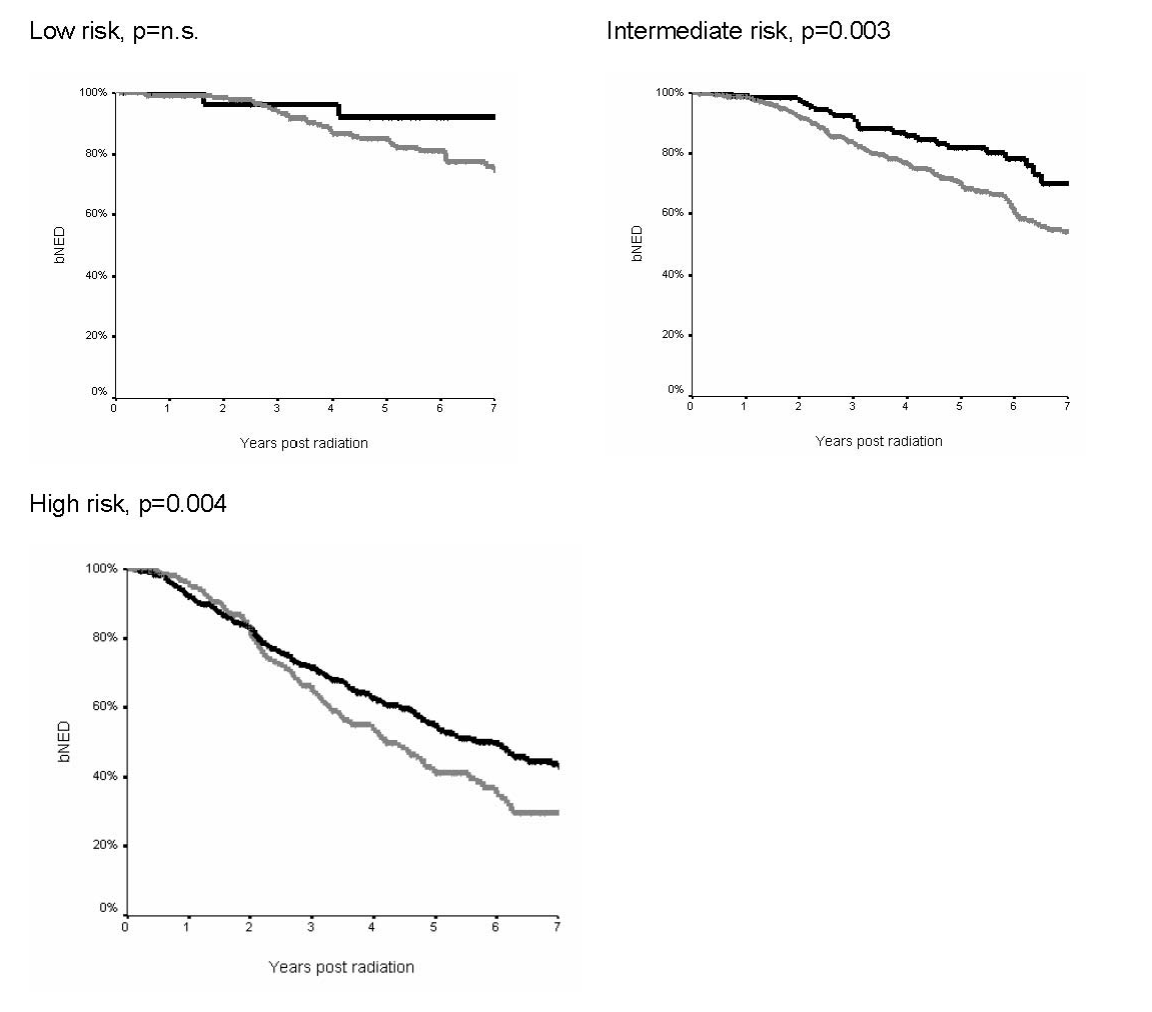
**Kaplan Meier curve for bNED (biochemical lack of evidence of disease survival) according to NCCN risk groups [3].** Black lines show combined EBRT and ADT, grey lines EBRT alone. P values refer to the log-rank test.

The Kaplan-Meier survival curves for biochemical control by NCCN risk groups are presented in figure 1. In the low risk group there was no difference in 5 year bNED between the patients treated with ADT and without (92% versus 85% respectively, p = 0.33). In the intermediate risk group the 5 year bNED rate was significantly higher in the patients treated with ADT at 82% compared to those treated without at 70% (p = 0.003). In the high risk group there was also a difference between those treated with and without ADT, 55% versus 42% respectively (p = 0.004).

**Figure 2:**
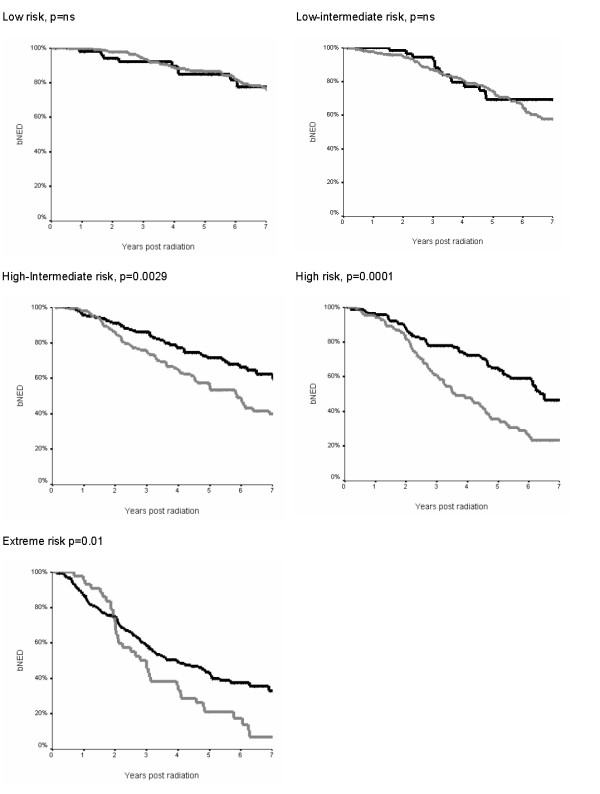
**Kaplan Meier curve for bNED (biochemical lack of evidence of disease survival) according to expanded risk group [6]**. 5-year bNED rates and hazard ratios are in Table 3. Black lines show combined EBRT and ADT, grey lines EBRT alone. P values refer to the log-rank test.

With the expanded 5-risk grouping significant differences in bNED was seen in the high-intermediate, high and extreme risk groups but not the low and low-intermediate risk groups (figure 2 and table 3). For the low intermediate group 5 year bNED survival was 75% with ADT and 70% without (p = 0.43). For the high intermediate group 5 year bNED survival was 72% with ADT and 55% without (p = 0.0025). There was no significant effect on cancer specific survival or overall survival for any group.

**Table 3:** 5-year biochemical lack of evidence of disease (bNED) in patients treated for prostate cancer with EBRT sorted by 5-level risk stratification, with the hazard ratio of relapse with/without ADT and corresponding p values are generated from the Kaplan-Meier log-rank test.

Risk Group	With ADT	Without ADT	Hazard ratio [95% CI]	P value
Low	84%	86%	1.8 [0.2–16.6]	n.s
Low intermediate	75%	70%	1.1 [0.56–2.1]	n.s
High intermediate	72%	55%	0.57 [0.4–0.83]	P = 0.0029
High	64%	36%	0.47 [0.33–0.69]	P = 0.0001
Extreme	43%	21%	0.61 [0.42–0.89]	P = 0.01

## Discussion

The use of androgen deprivation therapy in combination with EBRT has increased substantially over recent years, with, for example, data from the Cancer of the Prostate Strategic Urologic Research Endeavor (CAPSURE) in the United States suggesting that the rate of use has increased from 9.8% to 74.6% between 1989–1990 and 2000–2001[12]. This is undoubtedly related to several large randomized clinical trials which have shown a benefit to the combined treatment. This increased usage is paralleled by substantial toxicity related to castrate physiology, with physical, psychological and sexual side effects often being detrimental to a patient's quality of life. With these issues in mind, we demonstrated that there is potential to improve the selection of patients for treatment with combined hormonal manipulation based on contemporary knowledge of outcome prognostication.

To date, accurately identifying which patients will benefit from the addition of ADT to EBRT has been difficult, due to inter-trial differences in factors such as the stage and grade of the patients, dose and volume of radiation and the duration and timing of ADT. In terms of the tumour characteristics, most early studies have focused on patients with locally advanced disease. The Radiation Therapy Oncology Group (RTOG) 86–10 study [13] randomized patients with bulky primary cancers to either 2 months neoadjuvant and 2 months concurrent ADT with EBRT or EBRT alone. There was improved bNED and local control at 8 years with the addition of ADT, and the subgroup with Gleason score 2–6 cancers (many notably lacked baseline PSA levels) had a statistically improved overall survival at 70% compared to 52% with EBRT alone. Similarly, locally advanced (T3–4) or node positive cancers were included in EORTC 22863 [1] and RTOG 85–31 [14] trials, and randomized to have either immediate long term ADT starting at EBRT completion, or observation with delayed ADT. Overall survival was improved from 62% to 78% at five years in the EORTC trial (p = 0.0002), and improved in the Gleason score 8–10 subset of the RTOG trial. The Trans-Tasman Radiation Oncology Group (TROG) 96.01 trial of locally advanced prostate cancer [2] showed a benefit of adding ADT in terms of local failure, bNED, disease free survival and freedom from salvage treatment for both 3 and 6 months of neoadjuvant ADT.

Possibly more relevant to contemporary cohorts, the trial by D'Amico et al [15] randomized 206 patients with T1b – T2b tumors with a Gleason score greater than 6 to either EBRT (70 Gy) alone or EBRT with 6 months ADT. After an average follow-up of 4.5 years the estimated overall survival at 5 years was 88% for the combined treatment arm compared to 78% for radiotherapy alone (p = 0.04). There were only 6 prostate-cancer deaths in this trial, and given the small patient numbers the results should be treated with caution until replicated. Overall however, the general evidence appears to be in favour of the addition of ADT to EBRT in many prostate cancers, and a variety of ADT durations have resulted in significant gains.

Logically, the duration of ADT has since become the focus in a number of studies. A Canadian study which included T1c-T4 tumors, compared 3 and 8 months neo-adjuvant ADT and showed improvements in cause-specific survival in a high risk sub-set with longer ADT durations, according to a recent oral update of a prior publication [16]. The RTOG 92–02 study looked at more prolonged ADT, comparing 4 months with 24 months. This showed improvements in local control, bNED, cause-specific survival and freedom from distant metastases with prolonged ADT but an overall survival benefit was only seen for those with Gleason score 8 – 10. This benefit, curiously, was only seen in those with community-generated pathology reports, and was no longer present after central review[17]. TROG 96.01 also suggested cancer specific survival was also improved with 6 months ADT rather than zero or three months [2]. A further large TROG study ("RADAR") comparing 6 with 18 months ADT has finished accrual. The net result of these mixed studies is that ADT is used almost universally in high risk cancers, and also for intermediate risk in many centres. Using these standard NCCN criteria our data further reinforces these findings. Additionally, we suggest that by using an alternative risk stratification, the bNED benefit of additional ADT is confined to the high-intermediate, high and extreme risk groups.

No significant effect on cancer specific or overall survival was able to be demonstrated. This may be because the follow-up is too short and/or it may reflect the relatively high short-term efficacy of salvage ADT. Another possible explanation could be that the ADT duration for the high and extreme risk groups was insufficient. Prolonging the duration of ADT appears to benefit patients with a higher chance of relapse following EBRT [18,19]. A previous analysis from our institution also demonstrated an advantage to prolonged, rather than shorter ADT duration (6 months versus 12 versus 24 months) in patients with localized disease and a PSA above 20 [20]. In our current study however, the mean durations of ADT for the high and extreme risk groups were 15 months and 17 months respectively and the median duration of ADT treatment was only 11 months in both groups. However these criticisms do not affect our main conclusions, in that a bNED benefit is a necessary precursor to a survival benefit, and no bNED benefit was seen in the low-intermediate group.

The study presented here used prospectively collected data but a valid criticism of the comparison made is that it is not a randomized trial. There was likely case selection between those patients who received ADT and those who did not. A further issue is that the doses of radiation that were used, while typical for the era, are below contemporary levels. Whether or not dose-escalation beyond the doses used in the era of this study (66–70 Gy) would obviate any benefit of ADT in higher risk cancers is currently unknown, although subject to ongoing randomized trials. Improved bNED rates seen with escalated doses of radiation would suggest that the benefit might be less than that achieved with ADT. For example the absolute improvement in Phoenix bNED with 78 Gy versus 68 Gy in the Dutch randomized trial was 6% [21], which is substantially less than that observed from additional use of ADT in the present study for higher risk cancers where the bNED improvement was 18–28% depending on risk. Possibly, according to risk category, the strategies of dose escalation and ADT will work in different ways, and it would be logical to suppose that lower risk cancers have more to gain form dose escalation, and higher risk cancers from both ADT as well as dose escalation. Furthermore, previous trials of prolonged ADT in very advanced cancers have been criticized because they do not have an ADT-only arm. The National Cancer Institute of Canada PR3/Medical Research Council (UK) study, which randomized patients with locally advanced disease between ADT and ADT + EBRT, is yet to report, but should address this criticism.

ADT often causes significant side effects. During therapy, patients can suffer hot flashes, weight gain, gynaecomastia, impotence, loss of libido, fatigue and depression [22,23]. Longer-term side effects include a significant loss of bone mineral density after a year of ADT [24] and increased fracture rates with more prolonged durations of ADT[25]. A recently published study using the SEER database has demonstrated a significantly increased risk of developing incident diabetes, coronary heart disease, myocardial infarction and sudden cardiac death in men with prostate cancer who received ADT, when compared with those who did not [26] Even short durations of LHRH agonist therapy (1–4 months) were shown to carry increased risk of incident diabetes and coronary heart disease. D'Amico etc [27] has also shown increased fatal MI rates in those aged >65 years, with even short term ADT. There may also be adverse cognitive effects after 6 months of ADT [28]. Considering all these potential morbidities, ADT should be reserved for those with the highest chance of net benefit.

Overall, trials looking at the combination of ADT and EBRT have shown the greatest benefits for patients with locally advanced or high-grade tumors. This is reflected by our practice, in that higher risk patients were more likely to receive ADT. This was also the case in another retrospective review published recently [29]. The benefit of ADT in lower risk groups remains more controversial, and an improvement in bNED was not demonstrated in our dataset for low and low-intermediate risk patients. Therefore, these men (comprising 43%, of our cohort) may be safely spared the additional toxicity of ADT without compromising tumour control.

## Conclusion

This analysis divides the intermediate risk patients with localized prostate cancer and identifies the sub-groups who do, and do not obtain a bNED benefit from ADT. There appears to be no advantage to ADT where the Gleason score is 6 or less and PSA is 15 or less. ADT provides a bNED benefit in patients treated to standard dose radiation with Gleason 6 disease with a PSA greater than 15 or where the Gleason score is higher than 6.

## Competing interests

The authors declare that they have no competing interests.

## Authors' contributions

MB participated in the design of the study, checked data integrity, carried out the analyses and drafted the manuscript. SGW developed the new risk group system, participated in the design of the study and drafts of the manuscript. TP conceived of the study, co-developed the prospective data sources, participated in the design of the study and manuscript drafts. All authors have read and approved the final manuscript.
